# Acute Epiploic Appendagitis: An Overlooked Cause of Acute Abdominal Pain

**DOI:** 10.7759/cureus.10715

**Published:** 2020-09-29

**Authors:** Zainab Qudsiya, David Lerner

**Affiliations:** 1 Internal Medicine, Gulf Medical University, Ajman, ARE; 2 Department of Radiology: Abdominal Imaging, University of Washington, Seattle, USA

**Keywords:** epiploic appendagitis, acute abdomen, left lower quadrant pain, left iliac fossa pain, diverticulitis mimic, rare cause of acute abdominal pain, left-sided abdominal pain

## Abstract

Acute epiploic appendagitis is a benign condition caused by inflammation of the epiploic appendages that are serosal lined outpouchings of the colon lying adjacent to the tenia coli. This rare condition has non-specific clinical findings and is frequently misdiagnosed as either acute diverticulitis or acute appendicitis. However, unlike other surgical causes of acute abdomen, epiploic appendagitis is a self-limited condition and resolves with conservative management. CT of the abdomen plays a vital role in diagnosing this condition and excluding other causes of acute abdomen. This case report highlights the importance of being aware of this rare condition and its consideration in the differential diagnosis of acute lower abdominal pain to avoid unnecessary hospitalization and surgery.

## Introduction

Acute epiploic appendagitis is a self-limited condition characterized by inflammation and ischemic necrosis of the epiploic appendages secondary to torsion or thrombosis of the draining veins. Epiploic appendagitis typically presents with acute lower abdominal pain and localized tenderness in an afebrile well-looking patient. As the clinical presentation is non-specific, epiploic appendagitis is usually confused with other conditions such as acute appendicitis and acute diverticulitis. A study conducted by Rao et al. found that 11 out of 660 subjects (2%) with an initial clinical diagnosis of acute diverticulitis or acute appendicitis had CT findings diagnostic of epiploic appendagitis. Eight out of the eleven patients with epiploic appendagitis (72%) were initially misdiagnosed and underwent unnecessary hospitalization or antibiotic therapy [1]. This highlights the importance of CT abdomen for accurate diagnosis of this condition. Unlike other surgical causes of acute abdomen, epiploic appendagitis is managed conservatively with anti-inflammatory drugs. Here, we describe a case of a 30-year-old male patient who presented with left lower quadrant pain secondary to epiploic appendagitis of the descending colon and had characteristic findings on CT abdomen.

## Case presentation

A 30-year-old male patient presented to the outpatient clinic with a three-day history of lower abdominal pain. The pain was localized to the left lower quadrant, aching, intermittent, non-radiating, with an intensity of 7/10. The pain was aggravated by movement, partially relieved by acetaminophen, and had gradually worsened over three days. He denied a history of fever, loss of appetite, vomiting, constipation, diarrhea, tenesmus, blood in the stools, dysuria, and increased urinary frequency. There was no history of recent travel or contact with sick persons. He was not on any regular medications, and his past medical history was unremarkable. Past surgical history was significant for laparoscopic cholecystectomy at 25 years of age.

On examination, the patient was afebrile and hemodynamically stable. His temperature was 35.5℃, heart rate was 60 beats/min, blood pressure was 120/88 mm Hg, and his respiratory rate was 16 breaths/min. The abdomen was soft and non-distended with audible bowel sounds. Palpation revealed tenderness in the left iliac fossa with mild voluntary guarding. No rebound tenderness or rigidity was noted. The liver and spleen were not palpable. The remaining systemic exam was unremarkable. A provisional diagnosis of acute diverticulitis was made based on the clinical presentation.

**Figure 1:**
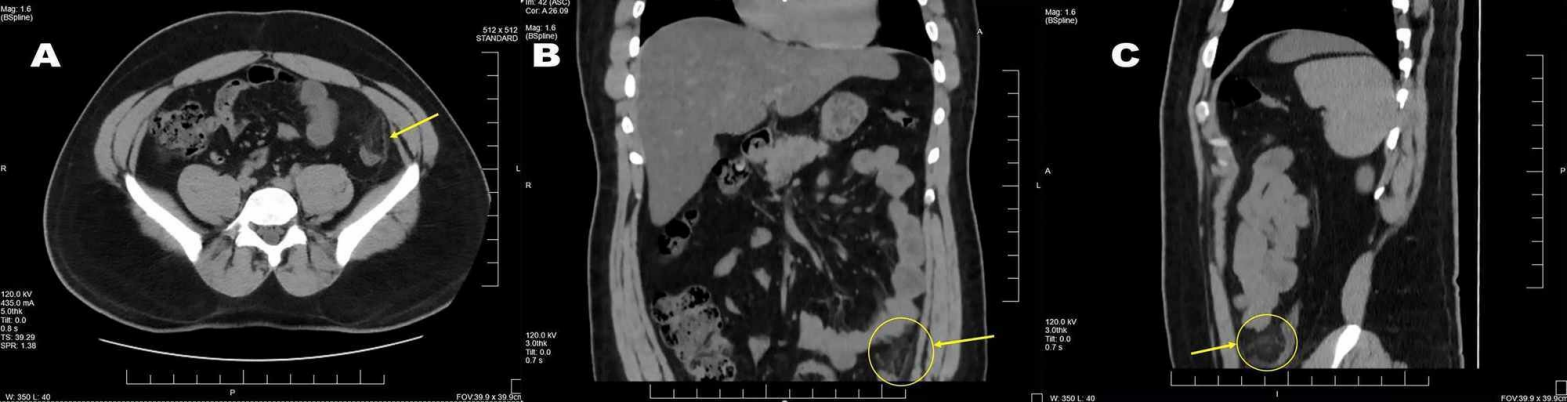
(A) Axial, (B) coronal, and (C) sagittal non-contrast CT images showing a fat-containing nodule adjacent to the descending colon with surrounding inflammatory stranding (arrows).

Lab investigations, including complete blood count, electrolytes, amylase, lipase, urinalysis, liver, and renal function tests, were within normal limits. A non-contrast CT of the abdomen and pelvis was ordered, which revealed a fat-containing nodule adjacent to the descending colon with surrounding inflammatory stranding, which was suggestive of acute epiploic appendagitis (Figure 1). The patient was discharged home in stable condition with ibuprofen for pain control. At a follow-up on day 5, the patient reported complete resolution of his pain with no recurrence.

## Discussion

Epiploic appendages are pouches lined by peritoneum arising from the antimesenteric border of the colon. These appendages contain adipose tissue, small circular veins, and arteries that supply the corresponding segment. Although their exact function is not known, studies suggest epiploic appendages provide mechanical support to the colon, aid in absorption, and also play an immunological role [2]. Epiploic appendages can be present along any part of the colon but are mainly concentrated around the transverse and sigmoid colon. Epiploic appendages can vary in size from 2 to 5 cm but can sometimes be as large as 15 cm. Long and large appendages are especially prone to torsion, which can occur at the pedicle and cause ischemic injury to the appendices. Ischemia of the appendices can also occur secondary to thrombosis of the draining veins. Ischemic injury leads to infarction and inflammation of the epiploic appendages, which is referred to as acute epiploic appendagitis.

Acute epiploic appendagitis is a rare condition with an incidence of 8.8 cases per million per year and occurs at a frequency of 1.3% [3]. This condition is more prevalent in men than in women, occurs more commonly in the third to fifth decades of life, and has a mean age at diagnosis of 40 years. It is also observed to occur more frequently in patients with a high percentage of visceral fat [4,5]. Epiploic appendagitis can affect any part of the colon but most commonly affects the sigmoid colon, followed by the descending colon, transverse colon, and the ascending colon [6].

Epiploic appendagitis typically presents with acute abdominal pain localized to either the left or right lower quadrant. The pain is described as a localized, dull, non-radiating, constant ache. Adherence of the necrotic appendage to the peritoneum can lead to exacerbation of pain with movement or deep inspiration [7]. In most cases, the pain is not associated with other symptoms. However, a minority of patients may report nausea, vomiting, diarrhea, postprandial fullness, or early satiety. On clinical examination, affected patients generally appear well, are vitally stable, and are afebrile. However, low-grade fever can be present in some cases. The most common positive clinical finding is localized abdominal tenderness with or without guarding. A third of patients may have a palpable abdominal mass. Rebound tenderness and rigidity are rare and should prompt consideration of an alternate diagnosis.

Lab investigations, including leucocyte count, are within normal limits in most cases. However, some patients may have mildly elevated inflammatory markers [8]. Radiological studies such as abdominal ultrasound and CT abdomen are required to identify this condition and exclude other causes of abdominal pain. On abdominal ultrasound, epiploic appendagitis appears as a non-compressible, ovoid solid hyperechoic mass surrounded by a hypoechoic rim [9]. The accuracy of ultrasound is user-dependent and varies depending on the amount of visceral fat in the patient. Therefore, CT abdomen is preferred over ultrasound and is the diagnostic test of choice. The most common CT finding is a fat dense ovoid lesion measuring approximately 1 to 5 cm seen adjacent to the anterior colonic wall and is present in almost 100% of cases. In 89% of cases, a hyperdense rim of inflamed visceral peritoneum is seen surrounding the ovoid mass. This finding is known as the *“*hyperattenuating ring sign*”* and is considered diagnostic of epiploic appendagitis [10]. Other radiologic findings include bowel wall thickening and disproportionate fat stranding [11], which occurs due to severe inflammation of the mesentery. *“*Central dot sign*”* refers to a high attenuation focus in the center of the inflamed appendage and occurs due to thrombosis of the draining appendageal vein. This finding is pathognomonic for epiploic appendagitis but is present only in 42% of cases. Therefore, its absence does not preclude a diagnosis of epiploic appendagitis. Chronically infarcted appendages may detach and may be visualized as calcified loose bodies in the peritoneal cavity [12,13].

Epiploic appendagitis mimics the clinical presentation of other conditions that can present with an acute abdomen. Right-sided epiploic appendagitis is usually misdiagnosed as acute appendicitis, whereas left-sided appendagitis is easily confused with acute diverticulitis. The presence of localized pain, as opposed to diffuse pain, helps to differentiate epiploic appendagitis from these conditions. Patients with epiploic appendagitis also lack other findings such as fever, vomiting, anorexia, rebound tenderness, and leukocytosis that are commonly present in acute appendicitis and diverticulitis. Another differential diagnosis for right-sided appendagitis is omental infarction. This self-limited condition affects the cecum and ascending colon and presents with right-sided abdominal pain. However, unlike acute epiploic appendagitis, this condition is more common in the pediatric population. In women, additional differentials include ovarian torsion, ovarian cyst rupture, and ectopic pregnancy. CT abdomen is an invaluable tool in excluding these differentials and confirming the diagnosis of epiploic appendagitis [14,15].

Acute epiploic appendagitis follows a self-limited course and usually resolves with conservative management within 3 to 14 days. Most patients can be safely managed at home with oral anti-inflammatory drugs such as ibuprofen. In patients with severe pain, a short course of oral opioids can be considered. Unlike other causes of acute abdominal pain, treatment with intravenous antibiotics and laparoscopy is unnecessary [16].

## Conclusions

This case illustrates a commonly overlooked cause of abdominal pain. Unlike other causes of acute abdomen, acute epiploic appendagitis is managed conservatively with anti-inflammatory drugs and has a self-limited course. Although it has non-specific clinical findings, the use of CT abdomen can help to diagnose this condition accurately and exclude other mimics. Knowledge of this rare condition and its characteristic radiological findings is essential to avoid unnecessary hospitalization, surgical intervention, and minimize the patient's financial burden.
